# Complete remission in refractory acute lymphoblastic leukemia using blinatumomab after failure of response to CD‐19 chimeric antigen receptor T‐cell therapy

**DOI:** 10.1002/ccr3.2918

**Published:** 2020-05-28

**Authors:** Francesco Paolo Tambaro, Sajad Khazal, Cesar Nunez, Dristhi Ragoonanan, Priti Tewari, Demetrios Petropoulos, Partow Kebriaei, William George Wierda, Kris Michael Mahadeo

**Affiliations:** ^1^ Department of Pediatrics Pediatric Stem Cell Transplantation and Cellular Therapy CARTOX Program The University of Texas MD Anderson Cancer Center Children's Cancer Hospital Houston Texas USA; ^2^ Bone Marrow Transplant Unit Pediatric Oncology Department AORN Santobono Pausilipon Naples Italy; ^3^ Department of Pediatrics The University of Texas MD Anderson Cancer Center Children's Cancer Hospital Houston Texas USA; ^4^ Department of Stem Cell Transplantation and Cellular Therapy CARTOX Program The University of Texas MD Anderson Cancer Center Houston Texas USA; ^5^ Department of Leukemia The University of Texas MD Anderson Cancer Center Houston Texas USA

**Keywords:** acute lymphoblastic leukemia, blinatumomab, chimeric antigen receptor

## Abstract

The T‐cell engager monoclonal antibody, blinatumomab, is a potential therapeutic strategy for refractory B acute lymphoblastic leukemia after failure of CD 19 chimeric antigen receptor T‐cell therapy.

## Graphical abstract

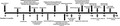

## To the Editor:

Curative options for patients with multiply relapsed/refractory B‐cell precursor acute lymphoblastic leukemia (B ALL) who fail novel treatments such as chimeric antigen receptor T‐cell therapy (CAR‐T) and the bispecific T‐cell engager monoclonal antibody, blinatumomab, remain dismal.^1^ The capacity of blinatumomab to recruit T cells to induce complete remission (CR) has been previously described.^2^ It is unclear whether the sequence of blinatumomab and CAR‐T administration results in competitive inhibition and impact outcomes, risk of antigen escape, and/or toxicity. Here, we report that blinatumomab is associated with disease remission in a patient with persistent disease following tisagenlecleucel CD19‐CAR‐T therapy.

**FIGURE 1:**
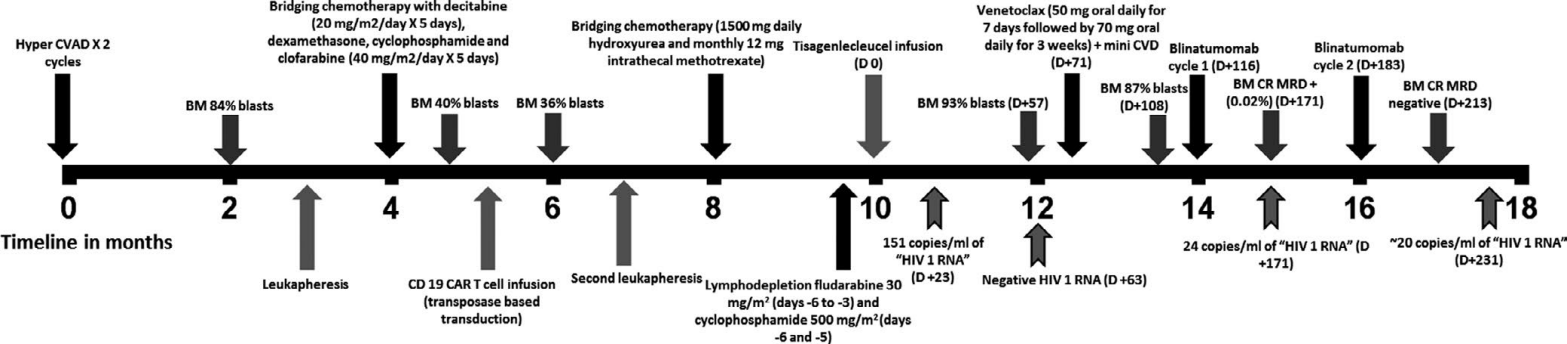
Seventeen‐year‐old male patient with multiply relapsed refractory B ALL. Clinical summary from presentation to complete remission

A 17‐year‐old male patient presented with history of B ALL, in third relapse, following second allo‐stem cell transplant. Aware of his prognosis and options to pursue palliative care, he elected to pursue further therapy with curative intent. His clinical course is summarized in Figure 1. He underwent autologous leukapheresis for CD19‐CAR‐T production (NCT02529813), and received bridging chemotherapy with decitabine, dexamethasone and fractionated cyclophosphamide; he then received clofarabine as continuing bridging chemotherapy, with sufficient leukemia control and no severe adverse events.

He received autologous CD19‐CAR‐T cells (1 × 10^6^ cells/kg) following lymphodepletion with fludarabine 25 mg/m^2^ and cyclophosphamide 250 mg/m^2^ (days −5 to −3).^3^ Neither immune effector cell‐associated neurotoxicity syndrome (ICANS) nor cytokine release syndrome (CRS) was observed. Subsequent disease evaluation showed persistent B ALL. He underwent a second leukapheresis and received bridging therapy with sufficient leukemia control and no severe adverse events.

He was subsequently treated with autologous CD19‐CAR‐T cells (tisagenlecleucel) (0.6 × 10^6^ cells/kg). Lymphodepletion consisted of fludarabine and cyclophosphamide. The patient's interleukin 6, C‐reactive protein, interferon gamma, and ferritin levels increased postinfusion; ICANs and/or CRS was not observed. Postinfusion bone marrow showed 93% blasts (CD19+, and negative for PD‐1, PDL‐1 expression).

He subsequently received venetoclax 50 mg oral daily for 7 days followed by 70 mg oral daily for 3 weeks in combination with mini‐hyper‐CVD^4^ with no response. He later received blinatumomab 5 µg/m^2^/d for 1 week, followed by 15 µg/m^2^/d for 3 weeks. The patient was hospitalized during this cycle of therapy for evaluation of fever in the setting of neutropenia. He had no hypoxia and no hypotension. All infectious studies were negative. This could represent cytokine release syndrome (maximum of grade 1) that did not require anticytokine therapy. Disease evaluation after the first cycle of blinatumomab (5 months post‐tisagenlecleucel infusion) showed complete hematological remission with 2% blasts in a hypocellular bone marrow and low minimal residual disease (MRD) (0.02%) by flow cytometry.

A second course of blinatumomab (15 µg/m^2^/d) was given and the patient ultimately achieved CR with negative MRD in bone marrow by flow cytometry and 100% 2nd donor chimerism demonstrated by FISH. He subsequently underwent a reduced intensity related haploidentical allo‐HSCT but died at 2 months post‐transplant from disseminated adenoviral infection.

As illustrated in Figure 1, ongoing pretransplant workup for our patient showed a positive human immunodeficiency virus‐1 (HIV) assay following CAR‐T administration, then a negative result around the time of B ALL disease assessment. Interestingly, a positive assay was again detected after blinatumomab administration around the time of CR achievement. Fourth generation combination (antigen and antibody) HIV testing was negative.

Innovative, potentially curative immunotherapeutic approaches for B ALL are based on (a) recruitment of autologous T cells with bispecific antibodies and (b) utilizing autologous T cells transduced ex vivo to express an engineered receptor that targets ALL cells. The negative MRD rates in pediatric ALL patients after CD19‐CAR‐T‐cell therapy vary from 81%to 90%, with event‐free survival and overall survival of 51.6% and 78%, respectively, at 6 months.^5^ In the pediatric population treated with CD19‐CAR‐T (tisagenlecleucel), the median time to maximum tisagenlecleucel expansion and median duration of persistence in the bloodstream were reported at 10 (range 5.7‐28) and 168 (range 20‐617) days, respectively.^5^

Mechanisms for nonresponse and loss of response have been described including (a) lack of expansion and short persistence of CD19‐CAR‐T, (b) antigen loss or low expression of CD19 on blasts, (c) high disease burden, and (d) increased expression of regulatory T cells (T_regs_).^6^

The therapeutic activity of blinatumomab is exerted through its capacity to recruit and activate T cells against CD19+ cells, resulting in negative MRD. Complete molecular remission was observed in 80% of treated MRD‐positive adult patients, while CR with and without hematologic recovery was reported in 43%‐44% and 39% negative MRD status in treated relapsed/refractory pediatric ALL patients.^7^ The potential for blinatumomab to engage and expand donor T cells to mediate an immune effector T‐cell response was reported in patients achieving negative MRD status and 100% donor T cells chimerism^2^ who underwent previous allo‐HSCT and in a patient receiving CD22‐CAR‐T Therapy.^8^ Interestingly, although patients who relapse after allo‐HSCT generally have poor outcome, improved survival was reported in relapsed/refractory pediatric patients who received allo‐HSCT prior to blinatumomab treatment (median overall survival 10.6 months vs 4.3 months).^9^ Blinatumomab binds CD19 through the murine IgG1 MoAb HD37^10^ while tisagenlecleucel binds through the single‐chain variable fragment (scFv) IgG2a MoAb FMC63^11^ which presents a different KD for the antigen. In our patient, complete molecular response may have been aided when blinatumomab synergistically targeted CD19 receptors with CAR‐T cells through the binding to two different CD19 epitopes. A sequential treatment with CD19‐CAR‐T cells followed by blinatumomab for persistent disease may be synergistic and overcome potential mechanisms of resistance. The epigenetic demethylation agent decitabine has been reported to increase expression of CD19 antigen and may increase efficacy of CD19‐CAR‐T cells.^12^ Bcl‐2 inhibitors may also enhance CD19‐CAR‐T efficacy in B‐cell malignancies in vitro.^13^ Our patient received both agents which may potentially explain the mechanism of CR. Prospective studies are needed to explore this further. There is no certified (Clinical Laboratory Improvement Amendments (CLIA)/(CAP) College of American Pathologists) assay for the detection of CAR‐T cells. Several reports have described lentiviral vector detection by use of COBAS AmpliPrep/COBAS TaqMan HIV‐1 Test, version 2, Roche Molecular Systems, Inc^14^, ^15^, ^16^ The lentivirus used in the manufacturing of tisagenlecleucel has overlapping sequences of transfer vector and primers with HIV‐1 used in this particular testing. The FDA package insert of the commercial tisagenlecleucel (KYMRIAH) indicates that some commercial HIV nucleic acid tests may yield false‐positive results in patients who have received this product. Among patients who achieved CR after tisagenlecleucel therapy, false‐positive assay results may correlate with presence of these CAR T cells.^14^

As new targeted therapies emerge for the treatment of B ALL, it is important to understand their potential interactions and determine the optimal sequence for treatment. Incremental reports in the literature may inform prospective studies, which may also explore potential synergy among such therapies.

## CONFLICT OF INTEREST

None declared.

## AUTHOR CONTRIBUTIONS

FPT and SK: completed the data collection and wrote the manuscript and share equal contribution. CN: treated the patient and assisted in editing the manuscript. DR: assisted in data collection. PT: assisted in editing the manuscript. DP and PK: treated the patient and assisted in editing the manuscript. WGW: assisted in editing the manuscript. KMM: treated the patient, assisted in analyzing the data and wrote the manuscript.
